# Analysis and control of the dynamical response of a higher order drifting oscillator

**DOI:** 10.1098/rspa.2017.0500

**Published:** 2018-02-21

**Authors:** Yang Liu, Joseph Páez Chávez, Ekaterina Pavlovskaia, Marian Wiercigroch

**Affiliations:** 1 College of Engineering, Mathematics and Physical Sciences, University of Exeter, Exeter EX4 4RN, UK; 2 Center for Applied Dynamical Systems and Computational Methods (CADSCOM), Faculty of Natural Sciences and Mathematics, Escuela Superior Politécnica del Litoral, PO Box 09-01-5863, Guayaquil, Ecuador; 3 Center for Dynamics, Department of Mathematics, TU Dresden, 01062 Dresden, Germany; 4 Centre for Applied Dynamics Research, School of Engineering, University of Aberdeen, Aberdeen AB24 3UE, UK

**Keywords:** vibro-impact drilling, position feedback control, progression optimization, bistability, chaos control

## Abstract

This paper studies a position feedback control strategy for controlling a higher order drifting oscillator which could be used in modelling vibro-impact drilling. Special attention is given to two control issues, eliminating bistability and suppressing chaos, which may cause inefficient and unstable drilling. Numerical continuation methods implemented via the continuation platform COCO are adopted to investigate the dynamical response of the system. Our analyses show that the proposed controller is capable of eliminating coexisting attractors and mitigating chaotic behaviour of the system, providing that its feedback control gain is chosen properly. Our investigations also reveal that, when the slider’s property modelling the drilled formation changes, the rate of penetration for the controlled drilling can be significantly improved.

## 1. Introduction

The adoption of the vibro-impact principle for drilling tools, known as downhole hammer, percussive hammer or percussive drills [1], has been used for construction, and later for oil and gas exploration, since the late 1940s. The operating principle of such a technique is that penetration can be achieved by repeatedly applying a large impulsive force to the drill-bit through a hydraulically or pneumatically operated piston impacting axially upon a drilling rod, and transferring the potential energy into kinetic energy of the drill-bit [2]. The merit of this mechanism is that rocks can be chipped and crushed easily by the impulsive force from the drill-bit, so that the rate of penetration (ROP) of the entire drill-string can be enhanced. Normally, vibro-impact drilling can significantly reduce wellbore creation time, and it is especially suitable for hard rocks [3]. In order to improve the performance of this technique, various drifting oscillator models [4–10], which can effectively predict the overall dynamics and progressive motion of vibro-impact drilling, have been studied in the past two decades. The main aim of these studies is to fully understand the dynamics of the drifting oscillators under various control parameters and to optimize their ROPs. Pavlovskaia *et al.* [4] studied the physical model of an impact system with a drift which can represent a number of practical driving tools, and revealed that the fastest penetration occurs when the system responds periodically. In [11], a simple control strategy was considered for this drifting system to improve its progression rates. This study suggests that the work which is done by the control forces must be positive in order to supply additional external energy to the system. In [5], an efficient semi-analytical method was developed for the drifting system to predict a range of control parameters for which the best progression rates were achieved. Luo & Lv [6] studied a two-degree-of-freedom plastic impact oscillator with a frictional slider, and the largest progression was observed when period-1 single-impact sticking motion with large impact velocity occurred. In [7], a vibro-impact moling rig, which was based on electro-mechanical interactions of a conductor with an oscillating magnetic field, was studied numerically and experimentally. Recently, modelling of high-frequency vibro-impact drilling was undertaken in [12]. In this study, a newly developed model of an existing experimental rig [3] was compared with the simplified low-dimensional model [4] which was created to describe the dynamic interaction between the drill-bit and the drilled formation. The best progression rates were identified through bifurcation analysis, and they were observed when the system response was periodic and the frequency of the response was the same as the frequency of the applied dynamic force. Until now, few works (e.g. [13]) have considered the optimization of vibro-impact drilling from a feedback control point of view, i.e. the question on how to best use system information as a feedback signal to improve the ROP and the stability of vibro-impact drilling remains open. This question defines the rationale of this paper, which considers using the displacement of the drill-bit as feedback to modulate the impulsive forces, and gives insight into its controlled dynamics for drilled rock formations.

Control of vibro-impact systems has been of great interest to the scientific community (e.g. [14–18]), and the main concern of these studies has been how to suppress chaotic motion and maintain the stability of the system under the noise present in the environment. In general, two non-smooth nonlinearities, namely impact and friction, are involved in these vibro-impact systems, which lead to their complexities in dynamics and sensitivities to external disturbances. Therefore, a control strategy must be in place to ensure their stabilities, particularly for their rich dynamical phenomena at near-grazing dynamics [19,20]. de Souza & Caldas [14] proposed a new procedure to implement the Ott–Gebogi–Yorke method to control the chaotic orbits in a mechanical system with impacts. Dankowicz & Jerrelind [15] studied a linear, discrete and closed-loop control strategy for ensuring the persistence of a local attractor in the near-grazing dynamics of an impact oscillator. In [16], a feedback control technique was applied to suppress chaotic behaviour in dissipative mechanical systems by using a small-amplitude damping signal. Later on, this technique was considered to control the chaotic motions of a number of vibro-impact and non-ideal oscillators [17]. Suppressing the bifurcation and chaotic-impact motions of a plastic impact oscillator was studied by Luo & Lv [18], using an external driving force, delay feedback and damping control law.

Apart from controlling chaos in vibro-impact systems, Liu *et al.* [21] studied the switching control between coexisting attractors for multi-stable vibro-impact systems. This was carried out by bringing the perturbed state of the system into the basin of attraction of a desired attractor using a short impulsive force [22]. The switching control ensures low power consumption of the system [23,24] yet maintains the system at some level of flexibility because its multi-stability is not affected. On the other hand, redundant coexisting periodic orbits must be suppressed if they can induce undesired performance or instability in the system, which means the switching control is invalid here and the multi-stable vibro-impact system needs to be converted into a monostable system. For example, a vibro-impact capsule system with forward and backward drifts presents multi-stability when the contact between the capsule and its supporting surface is sticky [25]. Here, the multi-stability of the capsule is manifested through a number of periodic orbits with low progression rates and a chaotic motion. So, a position feedback controller was designed to control the capsule to a monostable system with the desired direction of progression (i.e. forward or backward) [26,27]. In [8,28], coexisting attractors have been found for the drifting oscillator, and it was observed that multi-stability may affect the performance of vibro-impact drilling. Therefore, in this paper, we will investigate this phenomenon further and study its influence on the ROP of vibro-impact drilling.

The rest of the paper is organized as follows. In §2, the physical model and equations of motion of the higher order drifting oscillator are introduced, as well as the mathematical formulation of the position feedback control law. In §3, the proposed control method is studied numerically, and its capabilities in eliminating bistability, mitigating chaos and improving the ROP for different drilled formations are demonstrated through bifurcation analysis. Numerical investigation using the continuation methods, including one- and two-parameter analyses, is presented in §4. Finally, some concluding remarks are drawn in §5.

## 2. Mathematical modelling

**Figure 1.**
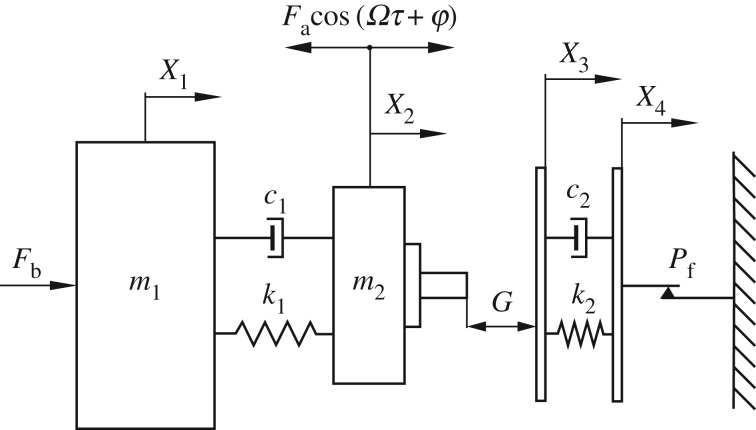
Physical model of the higher order drifting oscillator.

The vibro-impact drilling model considered in this investigation is shown in figure 1, which corresponds to a higher order drifting oscillator featuring soft impacts. The model includes a mass *m*_2_ representing the drill-bit assembly, which is driven by an external sinusoidal force with amplitude *F*_a_ and frequency *Ω*. This mass interacts with another element (mass *m*_1_) that accounts for all the components above the drill-bit assembly. A static force *F*_*b*_ is applied on *m*_1_, and the interaction between *m*_1_ and *m*_2_ is represented via a linear spring with stiffness *k*_1_ and a viscous damper with damping coefficient *c*_1_. The interaction between the drill-bit and the rock formation is modelled by a frictional, massless visco-elastic slider with stiffness and damping coefficients *k*_2_ and *c*_2_, respectively. The slider moves right in stick-slip phases, with the progression taking place when the force acting on the slider becomes larger than the threshold of the dry friction force *P*_f_. The variables *X*_1_, *X*_2_, *X*_3_ and *X*_4_ stand for the absolute position of the mass *m*_1_, drill-bit assembly *m*_2_, left slider plate and right slider plate, respectively. During operation, the condition *X*_2_−*X*_3_≥*G* is monitored, which is satisfied when the drill-bit assembly is in contact with the left plate of the frictional slider. Here, *G*>0 represents an initial gap between the drill-bit and the frictional slider.

The higher order drifting oscillator shown in figure 1 is a simplified version of the vibro-impact drilling system studied in [12]. In the present work, we group all the components above the drill-bit into a single mass (*m*_1_ in figure 1) in order to obtain a reduced form of the system considered in [12]. On the other hand, compared with the models studied in [4,29], there is an extra degree of freedom, due to the presence of the mass *m*_1_. The motivation for this is that the extra degree of freedom will allow us to understand the dynamics of the drill-bit under the constraint of other drill-string components, which provides a more realistic scenario than previous drifting oscillators of low dimension studied in the literature. In addition, the extra stiffness *k*_1_ introduces the possibility of studying in more detail the physical properties of the drilled media represented by the visco-elastic slider shown in figure 1, by varying the ratio between *k*_1_ and *k*_2_, which has not been investigated in the literature before.

### (a) Equations of motion

According to the mechanical set-up described previously, the higher order drifting oscillator can operate under any of the following regimes: *no contact*, *contact without progression* and *contact with progression*. The operation mode *no contact* occurs when *X*_2_−*X*_3_<*G*, i.e. the drill-bit and the rock formation are not in contact. In this case, the motion of the system is governed by the set of equations

2.1 $$\begin{array}{rl} & m_{1}{\ddot{X}}_{1}+c_{1}({\dot{X}}_{1}-{\dot{X}}_{2})+k_{1}(X_{1}-X_{2})=F_{b},\\ & m_{2}{\ddot{X}}_{2}+c_{1}({\dot{X}}_{2}-{\dot{X}}_{1})+k_{1}(X_{2}-X_{1})=F_{a}\;\cos\;(\Omega \tau +\varphi )+U_{p},\\ & c_{2}({\dot{X}}_{3}-{\dot{X}}_{4})+k_{2}(X_{3}-X_{4})=0,\\ & {\dot{X}}_{4}=0, \end{array}\}$$

where

2.2 $$U_{p}=K_{p}(X_{4}-X_{2})$$

is a position controller with control gain *K*_p_. This operation mode terminates when the mass *m*_2_ hits the left plate of the frictional slider (figure 1), which occurs precisely when *X*_2_−*X*_3_=*G*. After this, the system switches to one of the contact modes, *contact without progression* or *contact with progression*, depending on the force acting on the slider at the moment of contact. If this force does not exceed the dry friction threshold *P*_f_, the system switches to *contact without progression*, described by the system of ODEs

2.3 $$\begin{array}{rl} & m_{1}{\ddot{X}}_{1}+c_{1}({\dot{X}}_{1}-{\dot{X}}_{2})+k_{1}(X_{1}-X_{2})=F_{b},\\ & m_{2}{\ddot{X}}_{2}+c_{1}({\dot{X}}_{2}-{\dot{X}}_{1})+k_{1}(X_{2}-X_{1})+c_{2}({\dot{X}}_{3}-{\dot{X}}_{4})+k_{2}(X_{3}-X_{4})=F_{a}\;\cos(\Omega \tau +\varphi )+U_{p},\\ & X_{3}=X_{2}-G,\;{\dot{X}}_{3}={\dot{X}}_{2},\\ & {\dot{X}}_{4}=0. \end{array}\}$$

If the slider and the drill-bit are in contact, and the force acting on *m*_2_ from the slider becomes larger than the dry friction threshold *P*_f_, the system operates under the regime *contact with progression*, whose dynamics is governed by the equations

2.4 $$\begin{array}{rl} & m_{1}{\ddot{X}}_{1}+c_{1}({\dot{X}}_{1}-{\dot{X}}_{2})+k_{1}(X_{1}-X_{2})=F_{b},\\ & m_{2}{\ddot{X}}_{2}+c_{1}({\dot{X}}_{2}-{\dot{X}}_{1})+k_{1}(X_{2}-X_{1})+P_{f}=F_{a}\;\cos(\Omega \tau +\varphi )+U_{p},\\ & X_{3}=X_{2}-G,\;{\dot{X}}_{3}={\dot{X}}_{2},\\ & c_{2}({\dot{X}}_{3}-{\dot{X}}_{4})+k_{2}(X_{3}-X_{4})=P_{f}. \end{array}\}$$

### (b) Non-dimensionalization and variable transformation

In our investigation, we will use the following dimensionless variables and parameters:

2.5 $$\begin{array}{rl} \Omega_{0} & =\sqrt{\frac{k_{2}}{m_{2}}},\;\omega =\frac{\Omega }{\Omega_{0}},\;t=\Omega_{0}\tau ,\;a=\frac{F_{a}}{P_{f}},\;b=\frac{F_{b}}{P_{f}},\;\zeta =\frac{c_{2}}{2m_{2}\Omega_{0}},\\ g & =\frac{k_{2}}{P_{f}}G,\;\alpha =\frac{m_{2}}{m_{1}},\;\beta =\frac{k_{1}}{k_{2}},\;\gamma =\frac{c_{1}}{c_{2}},\;x_{1}=\frac{k_{2}}{P_{f}}X_{1},\;x_{2}=\frac{k_{2}}{P_{f}}X_{2},\\ x_{3} & =\frac{k_{2}}{P_{f}}X_{3},\;x_{4}=\frac{k_{2}}{P_{f}}X_{4},\;y_{1}=\frac{\text{d}x_{1}}{\text{d}t},\;y_{2}=\frac{\text{d}x_{2}}{\text{d}t},\;k_{p}=\frac{K_{p}}{k_{2}}. \end{array}\}$$

For numerical purposes, it is convenient to analyse the drifting oscillator as a piecewise-smooth dynamical system, which is a mathematical framework suitable for the application of path-following methods via the continuation platform COCO. Let us denote by $u:={\left(z_{1},w_{1},z_{2},w_{2},z_{3}\right)}^{T}\in R^{5}$ and $\lambda :=(\alpha ,b,\beta ,\gamma ,\zeta ,a,\omega ,k_{p},g,\varphi )\in R^{9}\times [0,2\pi )$ the state variables and parameters of the piecewise-smooth system, respectively. The state variables defined here are related to those introduced in (2.5) via the linear transformation

2.6 $$\begin{array}{rl} z_{1} & =x_{1}-x_{4},\\ w_{1} & =y_{1},\\ z_{2} & =x_{2}-x_{4},\\ w_{2} & =y_{2},\\ z_{3} & =x_{3}-x_{4}, \end{array}\}$$

which allows us to decouple the periodic behaviour of the system from the progression, as, for example, in [30]. In this setting, the vector fields to be used for the numerical implementation, after the transformations (2.5) and (2.6), are given by (one for each operation mode, see §2a) the following.

*No contact* (see equation (2.1)):

2.7 $$u^{\prime }=f_{\text{NC}}(u,\lambda ,t):=\left(\begin{array}{c} w_{1}\\ \alpha b-\alpha \beta (z_{1}-z_{2})-2\alpha \gamma \zeta (w_{1}-w_{2})\\ w_{2}\\ \beta (z_{1}-z_{2})+2\gamma \zeta (w_{1}-w_{2})+[a\cos(\omega t+\varphi )-k_{p}z_{2}]\\ -\frac{1}{2\zeta }z_{3} \end{array}\right),$$

where the prime denotes the derivative with respect to the non-dimensional time *t*.

*Contact without progression* (see equation (2.3)):

2.8 $$u^{\prime }=f_{\text{C1}}(u,\lambda ,t):=\left(\begin{array}{c} w_{1}\\ \alpha b-\alpha \beta (z_{1}-z_{2})-2\alpha \gamma \zeta (w_{1}-w_{2})\\ w_{2}\\ \beta (z_{1}-z_{2})+2\gamma \zeta (w_{1}-w_{2})-2\zeta w_{2}-z_{3}+[a\cos(\omega t+\varphi )-k_{p}z_{2}]\\ w_{2} \end{array}\right).$$

*Contact with progression* (see equation (2.4)):

2.9 $$u^{\prime }=f_{\text{C2}}(u,\lambda ,t):=\left(\begin{array}{c} w_{1}-w_{2}-\frac{1}{2\zeta }(z_{3}-1)\\ \alpha b-\alpha \beta (z_{1}-z_{2})-2\alpha \gamma \zeta (w_{1}-w_{2})\\ -\frac{1}{2\zeta }(z_{3}-1)\\ \beta (z_{1}-z_{2})+2\gamma \zeta (w_{1}-w_{2})-1+[a\;\cos(\omega t+\varphi )-k_{p}z_{2}]\\ -\frac{1}{2\zeta }(z_{3}-1) \end{array}\right).$$

In this mathematical framework, the system can be written in compact form as follows:

2.10 $$u^{\prime }=\left\{ \begin{array}{ll} f_{\text{NC}}(u,\lambda ,t), & h_{\text{IMP}}(u,\lambda )<0\text{ or }h_{\text{C1}}(u,\lambda )\leq 0,\\ f_{\text{C1}}(u,\lambda ,t), & h_{\text{IMP}}(u,\lambda )=0\text{ and }0<h_{\text{C1}}(u,\lambda )<1,\\ f_{\text{C2}}(u,\lambda ,t), & h_{\text{IMP}}(u,\lambda )=0\text{ and }h_{\text{C1}}(u,\lambda )\geq 1, \end{array}\right.$$

where

$$h_{\text{IMP}}(u,\lambda ):=z_{2}-z_{3}-g\;\text{and}\;h_{C1}(u,\lambda ):=2\zeta w_{2}+z_{3}$$

are event functions used to detect the transitions between the operation modes of the system.

Note that the dimension of the model has been reduced by 1 (see equations (2.1), (2.3) and (2.4)). This is because the progression (captured by the variable *x*_4_) of the system has been decoupled from the model. The progression, however, can be reconstructed from the system (2.10) as follows. Consider a solution *u*(*t*)=(*z*_1_(*t*),*w*_1_(*t*),*z*_2_(*t*),*w*_2_(*t*),*z*_3_(*t*))^*T*^ of (2.10), for *t*≥0. Then

$$x_{4}(t)=x_{4}^{0}+\int_{0}^{t}v(\tau )\;d\tau ,$$

where $x_{4}^{0}\in R$ represents an initial position at the beginning of the current progression phase, and

$$v(t)=\left\{ \begin{array}{ll} w_{2}(t)+\frac{1}{2\zeta }(z_{3}(t)-1), & h_{\text{IMP}}(u(t),\lambda )=0\text{ and }h_{\text{C1}}(u(t),\lambda )\geq 1,\\ 0, & \text{otherwise,} \end{array}\right.$$

which gives the velocity of the right plate of the slider for all *t*≥0 (see equations (2.1)–(2.6)). If, in addition, the solution *u*(*t*), *t*≥0, is periodic with period *T*_0_>0, the ROP can be computed as

$$\text{ROP}:=\frac{1}{T_{0}}(x_{4}(T_{0})-x_{4}^{0}),$$

which represents the average velocity of the right plate of the frictional slider shown in figure 1, during one period of motion.

## 3. Bifurcation analysis

In the following subsections, we will analyse the behaviour of the higher order drifting oscillator via monitoring the velocity of the mass *m*_2_, *y*_2_ and calculating the ROP.

### (a) Controlling bistability

**Figure 2.**
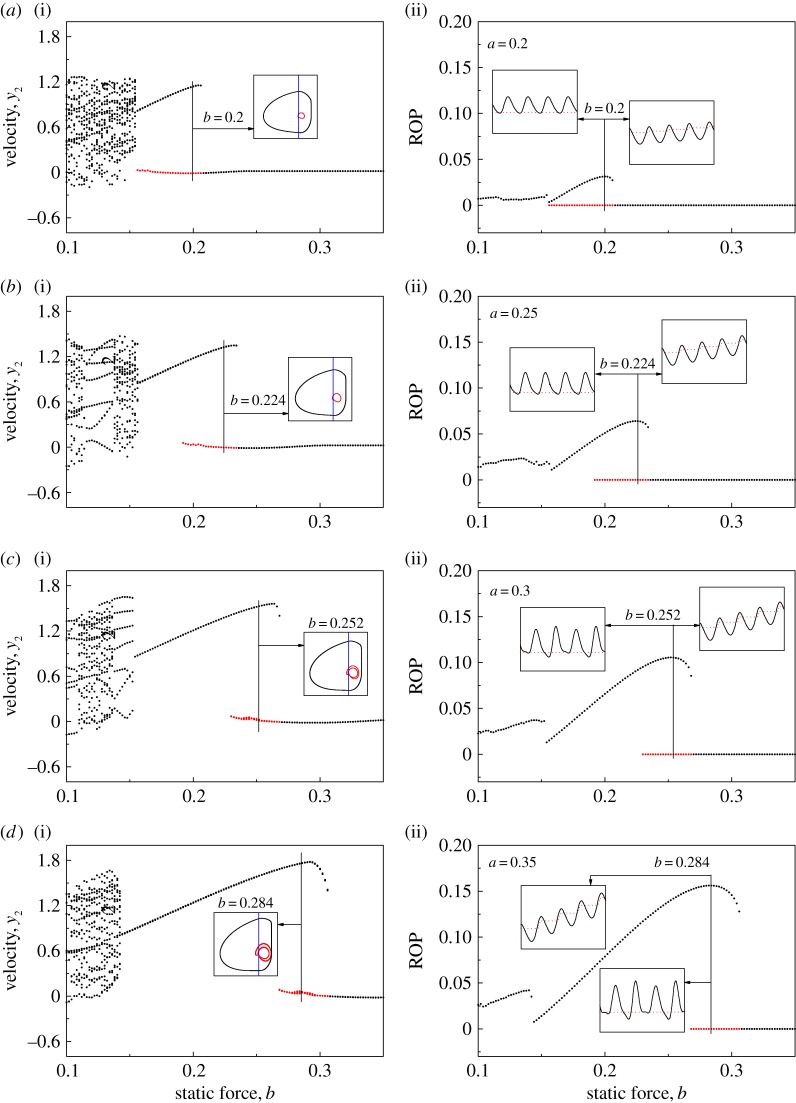
Bifurcation diagrams showing velocities (*a*(i)–*d*(i)) and ROPs (*a*(ii)–*d*(ii)) of the drifting oscillator calculated for *α*=0.1, *β*=0.1, *γ*=1.0, *ζ*=0.05, *g*=0.02, *ω*=0.53, *φ*=0, (*a*) *a*=0.2, (*b*) *a*=0.25, (*c*) *a*=0.3 and (*d*) *a*=0.35. Coexisting attractors are denoted by red dots in the bifurcation diagrams. The insets in *a*(i)–*d*(i) present the system trajectories on the phase plane (*x*_2_–*x*_4_, *y*_2_), and the locations of the impact surface are shown by bluelines. The insets in *a*(ii)–*d*(ii) show the time histories of displacements of the drill-bit *x*_2_ (black solid lines) and the slider bottom *x*_4_ (red dashed lines). (Online version in colour.)

Bistability of vibro-impact drilling has been observed by Pavlovskaia & Wiercigroch [30], Ajibose *et al.* [8] and Páez Chávez *et al.* [28], and it is clear that some of the coexisting attractors have higher progression rates than others. Figure 2 presents a series of bifurcation diagrams which show the main attractors of vibro-impact drilling with large amplitude coexisting with the attractors (red dots) with small amplitude, which have no penetration under variation of the static force *b*. The insets in figure 2*a*(i)–*d*(i) present the system trajectories on the phase plane (*x*_2_−*x*_4_, *y*_2_), and the locations of the impact surface, which represent the contact of the drill-bit and the left plate of the frictional slider, are shown by blue lines. The insets in figure 2*a*(ii)–*d*(ii) show the time histories of displacements of the drill-bit *x*_2_ (black solid lines) and the slider bottom *x*_4_ (red dash lines). As can be seen from figure 2*a*, bistable attractors exist for *b*∈[0.154,0.208] and the best ROP was recorded at *b*=0.2. This coexistence may cause drilling inefficiency, such that the state of the system hops from the main attractor with large amplitude to one with small amplitude due to external perturbations. As the amplitude of excitation *a* increases, ROP increases and this bistability can be observed at the regimes where the best ROPs were recorded, as shown in figure 2*b*–*d*. In other words, there is always the risk that vibro-impact drilling becomes inefficient when the system is operated at the regime of best ROP. Compared with the ROP of the low-order drifting oscillator studied in [4], where the best ROP was obtained when static force was approximately 50% of the amplitude of excitation, our calculations show that the required static force is larger, more than 80% of the amplitude of excitation. This is due to the fact that the extra degree of freedom, i.e. the components above the drill-bit assembly, causes reduced progression rates and the new optimum regime of the operating control parameters. It is also noted from [12] that the optimum static force there is 40–50% of the dynamic amplitude, so including other degrees of freedom has a significant influence on the dynamics of the vibro-impact drilling which could increase or decrease this ratio.

**Figure 3.**
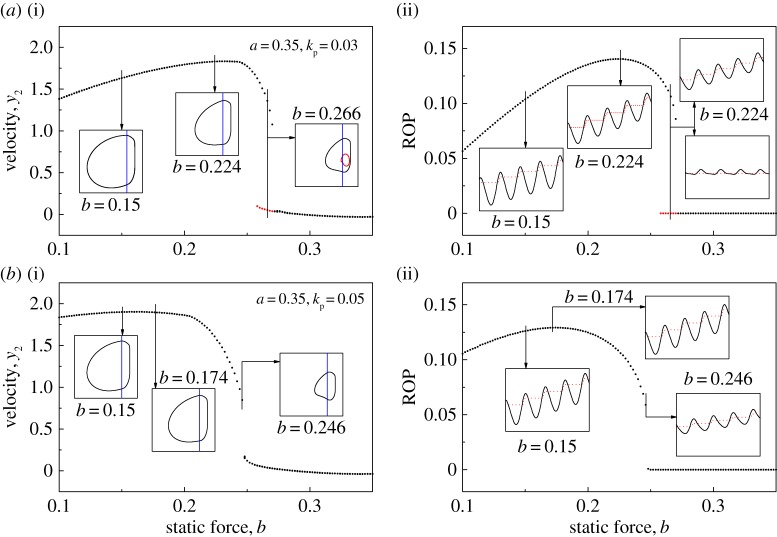
Bifurcation diagrams showing velocities (*a*(i),*b*(i)) and ROPs (*a*(ii),*b*(ii)) of the controlled drifting oscillator with (*a*) *k*_p_=0.03 and (*b*) *k*_p_=0.05 calculated for *α*=0.1, *β*=0.1, *γ*=1.0, *ζ*=0.05, *g*=0.02, *ω*=0.53, *φ*=0, *b*=0.2 and *a*=0.35. Coexisting attractors are denoted by red dots in the bifurcation diagrams. The insets in *a*(i),*b*(i) present the system trajectories on the phase plane(*x*_2_−*x*_4_, *y*_2_), and the locations of the impact surface are shown by blue lines. The insets in *a*(ii),*b*(ii) show the time histories of displacements of the drill-bit *x*_2_ (black solid lines) and the slider bottom *x*_4_ (red dash lines). (Online version in colour.)

Figure 3 shows the bifurcation diagrams when the position feedback controller was applied. It can be seen from figure 3*a* that, when *k*_p_=0.03, the regime of bistability has shrunk to *b*∈[0.258,0.272]. As the control gain *k*_p_ increases, the coexisting attractors disappear in figure 3*b*, and the system becomes monostable. However, the compromise is that the ROP of the drifting oscillator was reduced, and the best ROP was recorded at *b*=0.174.

### (b) Suppressing chaos

**Figure 4.**
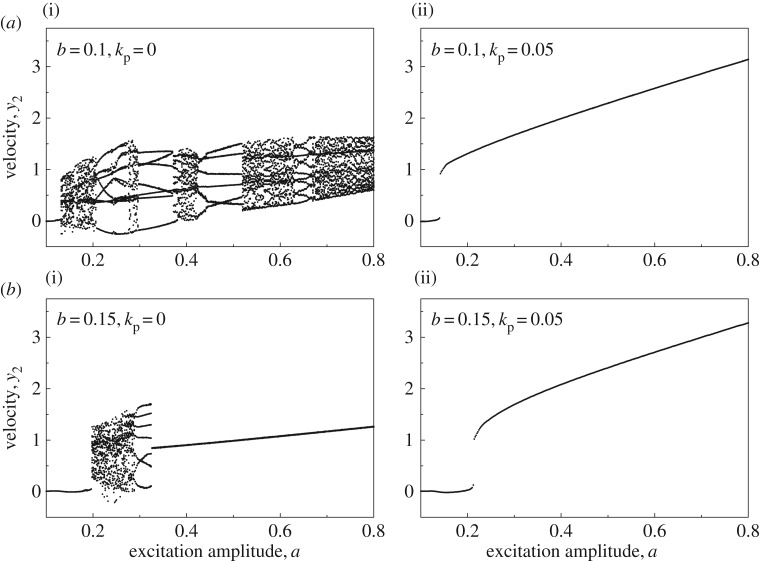
Bifurcation diagrams of the uncontrolled (*a*(i),*b*(i), *k*_p_=0) and the controlled (*a*(ii),*b*(ii), *k*_p_=0.05) drifting oscillator under variation of excitation amplitude *a* calculated for *α*=0.1, *β*=0.1, *γ*=1.0, *ζ*=0.05, *g*=0.02, *ω*=0.53, *φ*=0, (*a*) *b*=0.1 and (*b*) *b*=0.15.

**Figure 5.**
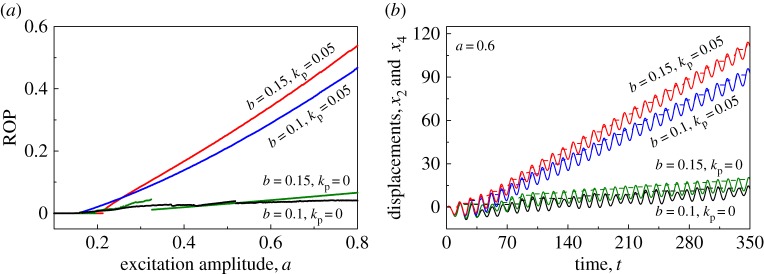
(*a*) ROPs for the uncontrolled and the controlled drifting oscillator under variation of excitation amplitude *a* calculated for *α*=0.1, *β*=0.1, *γ*=1.0, *ζ*=0.05, *g*=0.02, *ω*=0.53 and *φ*=0. (*b*) Displacements of the drill-bit (solid lines) and the slider bottom (dashed lines) obtained for *α*=0.1, *β*=0.1, *γ*=1.0, *ζ*=0.05, *g*=0.02, *ω*=0.53, *φ*=0 and *a*=0.6. (Online version in colour.)

It has been revealed in the literature (e.g. [4,12]) that insufficient static force *b* could lead to chaotic motion, causing instability of vibro-impact drilling. This has been shown in figure 4*a*(i),*b*(i) under variation of excitation amplitude *a*, when *b*=0.1 and *b*=0.15, respectively. When the controller was applied, chaotic motions were suppressed as demonstrated in figure 4*a*(ii),*b*(ii). ROPs before and after control at *b*=0.1 and *b*=0.15 are recorded in figure 5*a*, which indicates the efficacy of the position feedback controller. This example demonstrates the effectiveness of the proposed controller on improving the ROP of vibro-impact drilling when the static force is small. In order to show the dynamic behaviour of the system, figure 5*b* compares the time histories of displacements of the drill-bit and the slider bottom for *a*=0.6.

**Figure 6.**
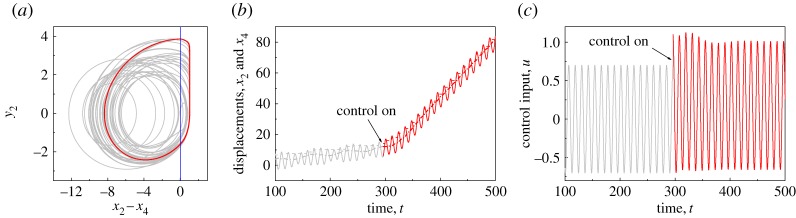
(*a*) Trajectory of the drifting oscillator on the phase plane (*x*_2_−*x*_4_, *y*_2_), (*b*) time histories of displacements of the drill-string *x*_1_ (dashed-dotted line), the drill-bit *x*_2_ (solid line) and the slider bottom *x*_4_ (dashed line), and (*c*) time histories of the control input *u* before and after the application of the position feedback controller obtained for *α*=0.1, *β*=0.1, *γ*=1.0, *ζ*=0.05, *g*=0.02, *ω*=0.53, *φ*=0, *a*=0.7 and *b*=0.1. The position feedback controller (*k*_p_=0.05) was switched on from the 26th period of external excitation, where *t*≈296.Grey and red lines represent the drifting oscillator before and after the application of the position feedback controller, respectively. The location of the impact surface, which indicates the contact of the drill-bit and the left plate of the frictional slider, is denoted by the blue line on the phase plane. (Online version in colour.)

Another example is presented in figure 6, where chaotic motion (grey lines) was observed initially, and the system response became the period-1 motion with one impact per period of excitation after the control was applied at *t*=296.39. It can be seen from figure 6*c* that the control input *u*, where $u=a\cos(\omega t+\varphi )+u_{p}$, was increased significantly due to the input of the position feedback controller *u*_p_. This can be interpreted as follows. When the control is not applied, a large static force may help to maintain the periodic motion of the drill-bit, but this stability will be lost once the static force becomes small. When the control is applied, it can preserve the stability of the drill-bit effectively. As can be seen from equation (2.2), when the distance between the drilled formation and the drill-bit (*x*_4_−*x*_2_) becomes large, the controller’s input *u*_p_ is large and more energy will be injected into the drill-bit so that it can impact and crush the formation efficiently. Another observation can be found from the trajectory of the drill-string (dashed-dotted line) shown in figure 6*b*, where harmful fluctuation of the drill-string was recorded initially, and it was stabilized when the control was applied.

### (c) Control of various slider properties

In practice, different formation properties may be encountered during the drilling process, and optimal excitation depends on the properties of the formation/slider [31]. So, it is desirable to have a control method which can maintain the best ROP at all times and which accommodates/adjusts to the changes in the drilled formation. In this section, we will demonstrate the capability of the proposed position feedback controller for retaining the ROP of vibro-impact drilling under various rock formations. It should be noted that, according to the mathematical model of the drifting oscillator used in this paper (figure 1), the parameters *β* and *γ* were altered to reflect the change of hardness in the rock formation, so the parameter *ζ* was affected accordingly. For example, if a 20% softer formation is considered, the new parameters will be *β*′=*β*/0.8 and *γ*′=*γ*/0.8, and the corresponding damping ratio becomes $\zeta^{\prime }=\zeta \sqrt{0.8}\approx 0.894\zeta$.

**Figure 7.**
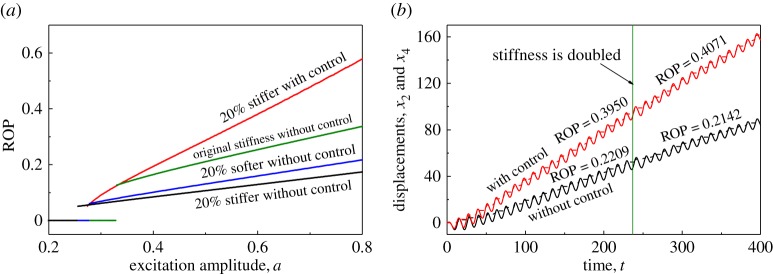
(*a*) ROPs for the uncontrolled and the controlled (*k*_p_=0.05) drifting oscillator under different rock mediums *k*_2_ and variation of excitation amplitude *a* calculated for black: *β*=0.083, *γ*=0.83, *ζ*=0.054; blue: *β*=0.125, *γ*=1.25, *ζ*=0.045; green: *β*=0.1, *γ*=1.0, *ζ*=0.05; red: *β*=0.083, *γ*=0.83, *ζ*=0.054, *α*=0.1, *g*=0.02, *ω*=0.53, *φ*=0 and *b*=0.25. (*b*) Time histories of displacements of the drill-bit *x*_2_ (solid lines) and the slider bottom *x*_4_ (dashed lines) for the system with and without control obtained for *α*=0.1, *β*=0.1, *γ*=1.0, *ζ*=0.05, *g*=0.02, *ω*=0.53, *φ*=0, *a*=0.6, *b*=0.25, *k*_p_=0 (black lines) and *k*_p_=0.05 (red lines). At *t*≈237.1, the hardness of the drilled formation is doubled,so the new parameters become *β*′=0.05, *γ*′=0.5, *ζ*′=0.07. (Online version in colour.)

Figure 7*a* presents the comparison between the ROPs obtained for the uncontrolled and the controlled drifting oscillator for various slider properties using the amplitude of excitation as a branching parameter. It can be clearly seen that, when the formation becomes 20% softer, the ROPs of drilling without control (blue line) reduce compared with the original drilled formation (green line). Such a decrease becomes worse when the drilled formation is 20% stiffer (black line) than the original one. Once the control had been applied, the ROPs for 20% stiffer formation were improved significantly. A further demonstration of the efficiency of the proposed controller is displayed in figure 7*b*, where displacements of the drill-bit and the slider bottom for the drifting oscillator with (red lines) and without (black lines) control are presented. As can be seen from the figure, the ROP of the drilling with control is 0.3950 and that without control is 0.2209. When the hardness of the drilled formation is doubled to *t*≈237.1, the ROP with control increases to 0.4071, while that without control decreases to 0.2142.

**Figure 8.**
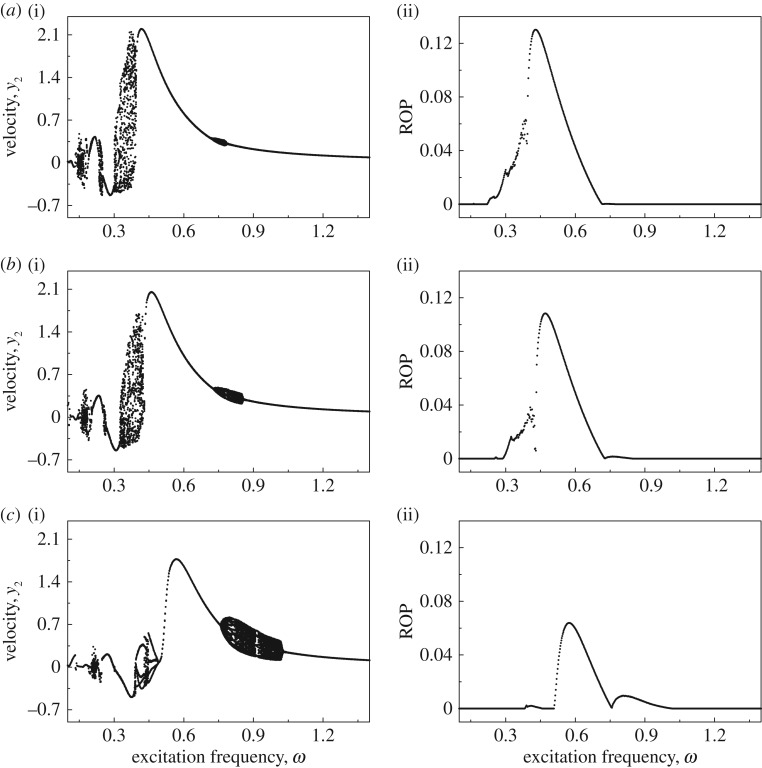
Bifurcation diagrams showing velocities (*a*(i)–*c*(i)) and ROPs (*a*(ii)–*c*(ii)) of the uncontrolled drifting oscillator under variation of excitation frequency *ω* calculated for *α*=0.1, *g*=0.02, *φ*=0, *a*=0.35, *b*=0.2 with (*a*) original stiffness: *β*=0.1, *γ*=1.0, *ζ*=0.05, (*b*) 20% softer: *β*=0.125, *γ*=1.25, *ζ*=0.045 and (*c*) 50% softer: *β*=0.2, *γ*=2.0, *ζ*=0.0354.

Bifurcation diagrams and ROPs as functions of excitation frequency *ω* of the uncontrolled drifting oscillator under various drilled formations are shown in figure 8. For the original drilled formation, the best ROP was recorded at *ω*=0.428. When the drilled formation becomes 20% softer, the best ROP was observed at *ω*=0.47. For both scenarios, it can be seen from figure 8*a*,*b* that the frequencies for the best ROPs are very close to the regimes of chaotic motions. So, it is very likely that vibro-impact drilling is led to chaos due to perturbations or external disturbances when it is operated under the chosen excitation for the best ROP. When the drilled formation becomes 50% softer, this issue improves, i.e. the optimum frequency for the best ROP is far from the regime of chaotic motion, but the frequency range for periodic motion is still small.

**Figure 9.**
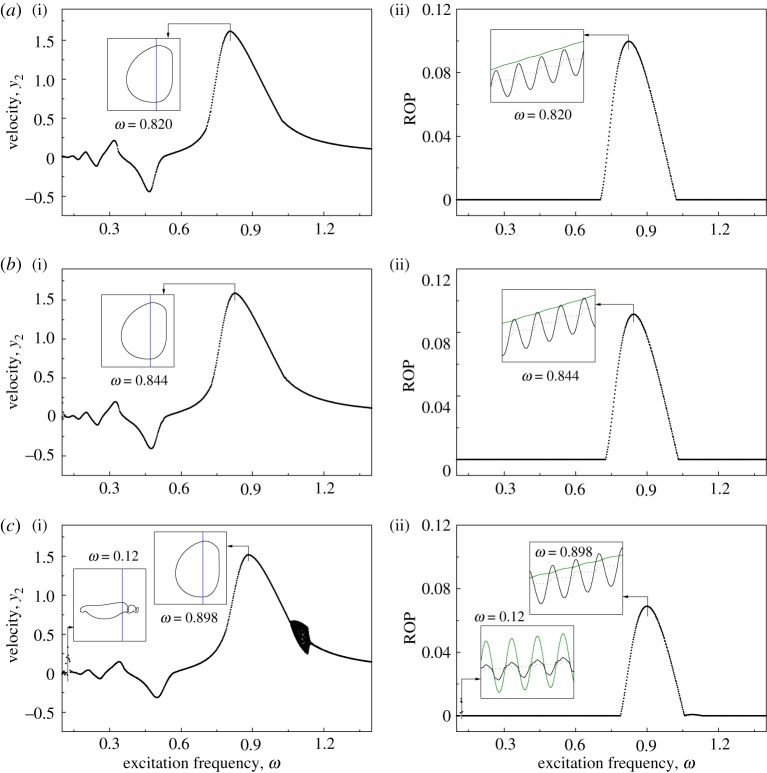
Bifurcation diagrams showing the velocities (*a*(i)–*c*(i)) and ROPs (*a*(ii)–*c*(ii)) of the controlled (*k*_p_=0.3) higher order drifting oscillator under variation of excitation frequency *ω* calculated for *α*=0.1, *g*=0.02, *φ*=0, *a*=0.35, *b*=0.2 with (*a*) original stiffness: *β*=0.1, *γ*=1.0, *ζ*=0.05, (*b*) 20% softer: *β*=0.125, *γ*=1.25, *ζ*=0.045 and (*c*) 50% softer: *β*=0.2, *γ*=2.0, *ζ*=0.0354. Insets in *a*(i)–*c*(i) present the trajectory of the oscillator on the phase plane (*x*_2_–*x*_4_, *y*_2_), and the insets in *a*(ii)–*c*(ii) show the time histories of displacements of the drifting oscillator. *x*_1_, *x*_2_ and *x*_4_ are denoted by green, black solid and black dashed lines, respectively. (Online version in colour.)

Figure 9 presents the bifurcation diagrams and ROPs for a controlled drifting oscillator (*k*_p_=0.3) under variation of excitation frequency *ω*. It can be seen that most of the chaotic motions have been suppressed, and the only chaotic regime recorded is *ω*∈(1.06,1.158) for 50% softer drilled formation, where no penetration has been observed. The insets in figure 9 show the trajectories and displacements of the drilling at some of the frequencies presenting the best ROPs. The frequencies for the best ROPs at the original, 20% softer and 50% softer levels were recorded at *ω*=0.820, 0.844 and 0.898, respectively. It is interesting to see that, at low frequency *ω*∈(0.1152,0.125), the controlled system has made some progression. However, comparing the insets in figure 9*c*, it is found that drill-string *m*_1_ (green line) has serious axial oscillations when the property of the slider is 50% softer, while drill-string *m*_1_ at other peak ROPs, e.g. *ω*=0.820, 0.844 and 0.898, progresses smoothly with drill-bit *m*_2_. Thus, this oscillating regime, which could cause drill-string instability, should be avoided when the property of the slider becomes softer.

**Figure 10.**
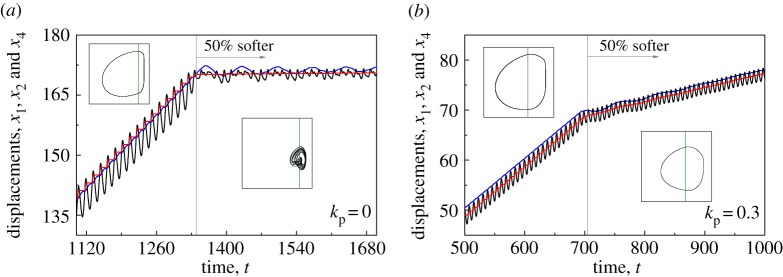
Time histories of displacements of *m*_1_ (blue lines), *m*_2_ (black lines) and the slider bottom (red lines) for the drifting oscillator (*a*) without control *k*_p_=0 at *ω*=0.428 and (*b*) with control *k*_p_=0.3 at *ω*=0.82 obtained for *α*=0.1, *g*=0.02, *φ*=0, *a*=0.35, *b*=0.2. The oscillator was operated under the original stiffness: *β*=0.1, *γ*=1.0, *ζ*=0.05, and after a number of periods of motion, the stiffness of the slider property became 50% softer: *β*=0.2, *γ*=2.0, *ζ*=0.0354. Insets present the trajectories of the oscillator before and after the stiffness of the slider property changed on the phase plane (*x*_2_−*x*_4_, *y*_2_). The location of the impact surface, which indicates the contact of the drill-bit and the left plate of the frictional slider, isdenoted by the green line on the phase plane. (Online version in colour.)

Figure 10 analyses the performance of the position feedback controller when the slider properties change. As can be seen from figure 10*a*, the drifting oscillator is operated using the optimum frequency *ω*=0.428, without applying the position controller. When the slider becomes 50% softer, the motion of the drifting oscillator varies from period-1 motion to aperiodic, and the progression rate drops drastically. When the controller is applied (*k*_p_=0.3) as shown in figure 10*b*, although the ROP of the drifting oscillator reduces, the periodic motion of the drifting oscillator is not affected significantly. This can also be observed from the trajectories of drill-string *m*_1_, which show that, when the property of the slider varies, the uncontrolled drill-string fluctuates greatly, while the controlled drill-string behaves following a period-1 response. The numerical observations reveal the effectiveness of the position feedback controller from a practical point of view, as it allows the operator of a vibro-impact drilling rig to stabilize the system response to an operation mode with a meaningful ROP, even when the properties of the drilled medium change, as shown in our numerical investigation.

## 4. Analysis of the system response via path-following methods

In this section, we will present a detailed numerical investigation of the dynamical response of the vibro-impact drilling model given by equation (2.10). For this purpose, we will apply numerical continuation methods for non-smooth dynamical systems, implemented via the continuation platform COCO [32,33]. Specifically, we will concentrate on the periodic response of the model observed in figure 2, which reveals the presence of coexisting attractors in the system. As can be seen in that figure, one attractor corresponds to a system behaviour for which the ROP is zero, while the other solution gives a non-zero ROP. In this section, we will determine whether the control method proposed in our study is able to eliminate this bistability, in such a way that an undesired transition from a progressing motion to an operation mode with zero ROP can be avoided.

### (a) One-parameter analysis

**Figure 11.**
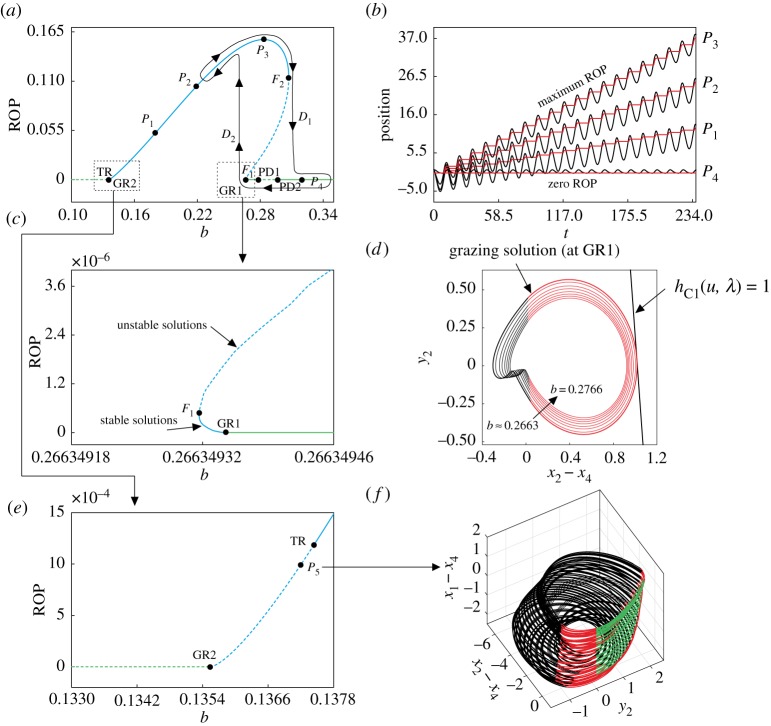
(*a*) Numerical continuation of the periodic orbit shown in figure 2*d* (in black) with respect to the static force *b*, computed for the parameter values *α*=0.1, *β*=0.1, *γ*=1, *ζ*=0.05, *a*=0.35, *ω*=0.53, *φ*=0, *g*=0.02 and *k*_p_=0 (no control). The points TR, F_*i*_ GR*i* and PD*i* represent the torus, fold, grazing and period-doubling bifurcations of limit cycles, while the labels P_*i*_ denote test points along the bifurcation diagram. The curve *D*_1_–*D*_2_ shows schematically a hysteresis loop of the system. (*b*) Time histories of the position of the mass *m*_2_ (*x*_2_, black) and the right plate of the frictional slider (*x*_4_,red), computed at the test points P_*i*_ shown in panel (*a*). (*c*) Blow-up of the bifurcation diagram depicted in panel (*a*) around the grazing bifurcation GR1. (*d*) Family of periodic orbits computed near the grazing bifurcation GR1. The orbits are plotted with black and red colours that represent the modes *no contact* and *contact without progression*, respectively. The straight line indicates the discontinuity boundary *h*_*C*1_(*u*,λ)=1, which defines the transition from *contact without progression* to *contact with progression*. (*e*) Enlargement of the boxed region around the point GR2 shown in panel (*a*). (*f*) Quasi-periodic solution of the system near the torus bifurcation TR, computed at the test point *P*_5_. The black and red colours are as in panel (*d*). In addition, green is used to mark the segments for which the system operates under the *contact with progression* mode.

The starting point for our study via path-following methods is the periodic solution plotted in figure 2*d* (in black), which corresponds to a system response with a positive ROP. In this case, the solution comprises the three operation modes described in the previous section: *no contact*, *contact without progression* and *contact with progression*. In figure 11*a*, we present the result of the numerical continuation of this orbit with respect to the static force *b*. In this figure, changes in stability are detected, which are marked with solid (for stable solutions) and dashed (unstable solutions) lines. As can be seen in the figure, for low values of *b* there is a branch of unstable periodic orbits with an ROP equal to zero, which means that the *contact with progression* mode is not present. If the parameter increases, a grazing bifurcation GR2 is detected for *b*≈0.13554, after which the ROP becomes positive, due to the birth of a solution segment corresponding to the *contact with progression* mode in the periodic orbit. Figure 11*e* presents a blow-up of the bifurcation diagram near the bifurcation GR2. Here, it can be seen that for a somewhat larger value of the static force (*b*≈0.13744) a torus bifurcation TR is found. At this point, the periodic solution becomes stable, because a pair of complex-conjugate Floquet multipliers of the periodic solution cross the unit circle from the outside, in such a way that all non-trivial multipliers of the periodic orbit have modulus less than 1 after TR.

In figure 11*f*, a (stable) quasi-periodic solution is computed at the test point *P*_5_ (*b*=0.13720), a system response that is produced by the torus bifurcation TR. From this point, a large segment of stable periodic solutions is found, denoted by the solid blue curve in figure 11*a*. This solid line finishes at *b*≈0.30722, where a fold bifurcation is found (*F*_2_). At this point, an unstable solution branch is born (dashed blue curve), which becomes solid again at another fold bifurcation labelled *F*_1_ (*b*≈0.26634932). Here, the periodic orbit becomes stable and then undergoes a grazing bifurcation for *b*≈0.26634934. At this point, the mode *contact with progression* disappears from the periodic response; as a result the ROP becomes zero. The stable orbit with zero ROP persists for larger values of the static force *b*, until a period-doubling bifurcation PD1 is found at *b*≈0.27833. Here, a real Floquet multiplier leaves the unit circle through −1; as a result, the (period-1) orbit loses stability and a stable period-2 solution is born. The unstable period-1 branch (dashed green line) finishes at *b*≈0.29662 (PD2), where another period-doubling bifurcation is detected. At this point, the real Floquet multiplier goes inside the unit circle again (through −1), and the stable period-2 solution disappears, while the period-1 orbit becomes stable and remains so for larger values of static force (within the parameter range considered in our computations).

Note that, along the solid blue line shown in figure 11*a*, a series of test points is computed, at the values *b*=0.18 (*P*_1_), *b*=0.22 (*P*_2_), *b*≈0.28325 (*P*_3_) and *b*=0.32 (*P*_4_). The behaviour of the system at these points is presented in figure 11*b*, which shows the time histories of the position of the mass *m*_2_ (*x*_2_) and the right plate of the frictional slider (*x*_4_). As can be seen in figure 11*b*, the ROP varies from zero (at *P*_4_) to approximately 0.15642 (at *P*_3_), where the ROP achieves a maximum. On the other hand, a family of periodic orbits with zero ROP is plotted in figure 11*d*, for the parameter range 0.2663≤*b*≤0.2766. Note that during the contact modes it holds that *h*_IMP_(*u*,λ)=*z*_2_−*z*_3_−*g*=0 (see (2.10)), hence

$$\begin{array}{rl} h_{\text{C1}}(u,\lambda ) & =2\zeta w_{2}+z_{3}\\ & =2\zeta w_{2}+z_{2}-g\\ & =2\zeta y_{2}+(x_{2}-x_{4})-g=1 \end{array}$$

represents the discontinuity boundary (straight line in figure 11*d*) that defines the transition from *contact without progression* to *contact with progression*, as explained in §4. When a periodic orbit makes tangential contact with this boundary, a grazing bifurcation takes place, as shown in figure 11*d*, which corresponds to the grazing point GR1 displayed in figure 11*c*.

Another important feature of the bifurcation scenario observed in the system is that there is a parameter window defined by the fold bifurcations *F*_1_ and *F*_2_ in which two attractors coexist (bistability). One attractor corresponds to a periodic solution with ROP positive, lying on the solid blue line plotted in figure 11*a*. The other coexisting attractor is given by a system response with ROP zero. This second attractor, however, is divided into two types: one with period twice that of the external excitation (for *b* between the bifurcations PD1 and PD2) and one with the same period as the external excitation, obtained for *b* below PD1 and above PD2. The attractor on the solid blue line can be identified as a desirable solution from a practical point of view, as it yields a positive ROP. The other attractor, on the contrary, should be avoided, as it gives zero progression that implies an inefficient use of energy. This motivates the study in the next section, in which we will investigate whether the control scheme applied in this paper is able to eliminate the bistability.

### (b) Two-parameter analysis

Our numerical investigation has so far revealed the presence of various codimension-1 bifurcations that affect the behaviour of the system in different ways. In particular, the interplay between the fold bifurcations *F*_1_ and *F*_2_ gives rise to hysteresis in the system, which is schematically represented by the closed curve *D*_1_–*D*_2_ plotted in figure 11*a* and produced by the presence of two coexisting attractors for each *b* in a parameter window, as explained in the previous section. If, for instance, the system is set to yield the maximum ROP (found at point *P*_3_ shown in figure 11*a*), an external perturbation may produce an undesired jump to the coexisting attractor with zero ROP, lying on the green branch depicted in the figure. One mechanism to deal with such a situation would be to use the hysteresis loop to switch back to the attractor with maximum ROP, which would require decreasing the static force below the fold point *F*_1_ so as to jump to the solid blue curve and then increasing the parameter until the optimal point *P*_3_ is reached again.

**Figure 12.**
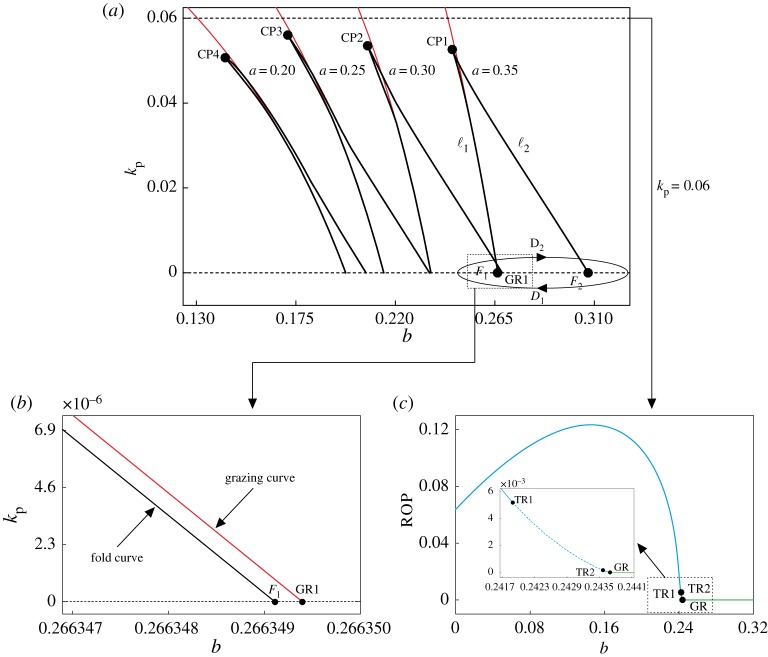
(*a*) Two-parameter continuationof the bifurcation points *F*_1_, *F*_2_ (both in black) and GR1 (red curve) shown in figure 11*a*, with respect to the static force *b* and the control gain *k*_p_. The resulting bifurcation diagram is computed for the cases *a*=0.35, 0.30, 0.25 and 0.20. The curve *D*_1_–*D*_2_ shows schematically the hysteresis loop plotted in figure 11*a*, obtained for *k*_p_=0 (no control). The points CP*i* denote a cusp bifurcation of limit cycles. (*b*) Blow-up of the bifurcation diagram in panel (*a*) around *F*_1_ and GR1. (*c*) One-parameter continuation with respect to the static force *b*. The parameter values are as in figure 11, except for *k*_p_=0.06. The inset depicts an enlargement of the boxed region.

In this section, we will investigate whether the control scheme applied to the vibro-impact drilling model can be used to eliminate the bistability, by suitably changing the control gain *k*_p_. In order to gain an insight into this matter we will carry out a two-parameter continuation of the codimension-1 points *F*_1_, *F*_2_ and GR1, found in figure 11*a*. These codimension-1 bifurcations play a fundamental role in the presence of bistability in the system, as will be seen in our numerical study. The result of the two-parameter continuation is presented in figure 12*a*, for the cases *a*=0.35, 0.30, 0.25 and 0.20. The labels *F*_1_, *F*_2_ and GR1 correspond precisely to the codimension-1 bifurcations detected in figure 11*a*, for *a*=0.35 and *k*_p_=0 (no control). Similarly, the curve *D*_1_–*D*_2_ shows schematically the hysteresis loop found previously. The labels ℓ_1_ and ℓ_2_ denote the locus of fold points obtained from the two-parameter continuation of the bifurcations *F*_1_ and *F*_2_, respectively. The numerical computations reveal the presence of a codimension-2 point (*b*,*k*_p_)≈(0.24561,0.05269) (CP1), where the twofold branches, ℓ_1_ and ℓ_2_, join together via a cusp singularity. This dynamical phenomenon explains the presence of the loop *D*_1_–*D*_2_ found in figure 11*a*, as such hysteretic effects are known to occur near a cusp point (see [34], §8.2). Further numerical computations indicate that the bifurcation scenario just explained is robust under small parameter variations, as its main qualitative features persist when the amplitude *a* is perturbed. In each case, a cusp singularity was found, for the values (*b*,*k*_p_)≈(0.20927,0.05391) (CP2), (*b*,*k*_p_)≈(0.17331,0.05603) (CP3) and (*b*,*k*_p_)≈(0.14543,0.05064) (CP4), when the amplitude of excitation is *a*=0.30, 0.25 and 0.20, respectively.

Another remarkable feature of the bifurcation picture described above is the closeness between the fold branch ℓ_1_ and the curve of grazing bifurcations plotted in red in figure 12*a*. A closer look at this is presented in figure 12*b*, which shows a blow-up of the two-parameter bifurcation diagram near points *F*_1_ and GR1. This is a dynamical scenario where a classical bifurcation (fold) is induced by a non-smooth bifurcation (grazing), which is a typical phenomenon observed in systems with soft impacts (cf. [35–37], example 2.3). On the other hand, note that when *k*_p_ increases from zero the size of the interval of bistability, defined by the fold curves ℓ_1_ and ℓ_2_, decreases. A critical point is reached when the horizontal line $k_{p}=k_{p}^{0}$, $k_{p}^{0}\geq 0$ reaches the cusp point CP1, above which the fold bifurcations defining the bistability disappear (figure 12*a*). Therefore, it can be seen that the control method applied to the vibro-impact drilling model is indeed capable of eliminating bistability by choosing a suitable control gain *k*_p_. Furthermore, this mechanism is robust under small parameter perturbations, as confirmed by the series of bifurcation diagrams obtained for several values of amplitude *a*. An example of the effectiveness of this approach to eliminate bistability is presented in figure 12*c*, where a one-parameter bifurcation diagram with respect to the static force *b* is computed for *k*_p_=0.06, above the cusp point CP1. Here, it can be seen that the hysteretic effects have been eliminated, and therefore the situation in which an undesired transition from an attractor with positive ROP to one with zero ROP is no longer possible, as in the case analysed in figure 11*a*, characterized by two coexisting attractors with positive and zero ROP, respectively.

## 5. Conclusion

We have studied a position feedback control strategy for controlling a higher order drifting oscillator with application to vibro-impact drilling. The causes of control issues for a drifting oscillator are twofold. Firstly, bistability has been observed when a drifting oscillator is operated in the optimum regime when the main attractor generating the best ROP coexists with the attractor with zero ROP. Secondly, the dynamics of the oscillator becomes chaotic when the static force (i.e. weight-on-bit) is applied insufficiently. In order to address these two issues, we proposed a position feedback controller which simply adopts the relative displacement between the mass *m*_2_ and the right slider plate, and our studies mainly focused on exploring its capability in improving the ROP and suppressing bistability and chaos by using path-following methods.

Our bifurcation analyses were carried out by using the static force, the frequency and the amplitude of excitation as branching parameters. For the scenario of using the static force as the branching parameter, we have observed that the system is always bistable when the optimum static force, which produces the fastest ROP, is applied regardless of the amplitude of external excitation. This coexistence may cause drilling inefficiency, such that the drilling system may experience state hopping from the main attractor with the fastest ROP to the attractor with zero ROP due to external perturbation. After applying the proposed controller, it was revealed that the system was converted from bistable to monostable, and the attractor with no progression was successfully removed. From our calculations, we found that the ROP for the controlled drifting oscillator decreased, but, as a compromise, the required static force providing the best ROP was significantly reduced.

For the scenarios of using the frequency and the amplitude of external excitation, we have demonstrated the effectiveness of the proposed controller in suppressing chaos caused by insufficient static force. Some examples were given to show the controlled dynamics of the drill-bit for a wide frequency range of external excitation. In addition, we have studied the position feedback controller under varying drilled formations, as the optimum set of control parameters will be affected when the drilled formation is changed. From the study, we have found that the ROP of the uncontrolled drifting oscillator decreases when the drilled formation becomes either softer or stiffer. When the control was applied, under the condition of varying drilled formation, the ROP of the system was significantly improved by appropriately choosing the feedback control gain.

To investigate the dynamical response of the drifting oscillator in detail, we applied numerical continuation methods for non-smooth dynamics systems, implemented via the continuation platform COCO. We focused on whether we can eliminate the bistability in such a way that an undesired transition from a progressing motion to a no progression one can be avoided. Based on our continuation studies, torus, grazing, fold and period-doubling bifurcations were identified in the drifting oscillator, which affect the behaviour of the system in different ways. From the one-parameter analysis, we found a parameter window defined by the fold bifurcations *F*_1_ and *F*_2_ in which bistability was detected. Here, one attractor corresponds to a periodic solution with positive ROP, and the other coexisting attractor is given by a system response with zero ROP. The second attractor can be further divided into two types: one with period twice that of the external excitation and one with the same period as the external excitation.

For the two-parameter analysis, our studies have focused on the interplay between the fold bifurcations *F*_1_ and *F*_2_ giving rise to the hysteresis in the system. We have followed the codimension-1 points *F*_1_, *F*_2_ and GR1, which play a fundamental role in the presence of bistability in the system. Our numerical computations have revealed the presence of codimension-2 points, where the twofold branches join together via a cusp singularity. Our computations have also indicated that the bifurcation scenario is robust under small parameter variations, as its main qualitative features persist when the amplitude of excitation is perturbed. Another remarkable finding of our two-parameter analysis is that, when the feedback control gain *k*_p_ increases, the size of the interval of bistability, defined by the fold curves, decreases, and the bistability disappears once the control gain reaches the cusp point. Therefore, this analysis allows us to identify the minimum control gain that guarantees the elimination of bistability.

## Supplementary Material

Code.zip

## Supplementary Material

mdl_kv.m

## Supplementary Material

run_mdl.m

## Data accessibility

Additional data are included in the electronic supplementary material.
